# Latent tuberculosis infection in China, 1990–2050: GBD-informed projections for age-targeted screening

**DOI:** 10.3389/fpubh.2025.1691385

**Published:** 2026-01-05

**Authors:** Qing-Qing Jiang, Xiao-Yu Zhang, Shao-Xian Li, Xiao Yu, You-De Liu, Wei Pan, Jian Xue

**Affiliations:** 1 Department of Hospital Infection Management, Qishan Hospital of Yantai, Yantai, China; 2 Department of Laboratory Medicine, Yantai Center for Disease Control and Prevention, Yantai, China; 3 Department of Tuberculosis Medicine and Treatment, Qishan Hospital of Yantai, Yantai, China; 4 AIDS Prevention and Control Section (APCS), Yantai Center for Disease Control and Prevention, Yantai, China

**Keywords:** latent tuberculosis infection, age-period-cohort analysis, disease burden, targeted screening, public health

## Abstract

**Objective:**

China bears high latent tuberculosis infection (LTBI) caseload, yet its longitudinal trends and age-specific burden remain unquantified, hindering targeted control.

**Methods:**

Leveraging data from the Global Burden of Disease Study 2021, we analyzed age-standardized LTBI prevalence rate (ASPR) and cases in mainland China (1990–2021). Joinpoint regression quantified temporal trends, while age-period-cohort (APC) modeling disentangled age/cohort effects. Bayesian age-period-cohort (BAPC) projected prevalence and cases to 2050.

**Results:**

From 1990 to 2021, China’s LTBI cases rose by 33.28% (1990–2021) despite declining ASPR (EAPC = −0.39). This contrasts with global trends. APC analysis indicated that the risk peak was more prolonged in mainland China, spanning the age range 25–60 years, while 20–29 years age globally. Over the next three decades, China’s prevalence will decline until 2036 (29,396.35/100,000), then rebound, reaching 30,184.31 by 2050. Cases peak earlier (2029: 0.42 billion) than globally (2036: 1.80 billion).

**Conclusion:**

Mainland China faces a uniquely prolonged LTBI risk window and an impending burden resurgence after 2036. Prioritizing age-targeted screening for 50–69-year-olds and strengthening surveillance are critical to achieving TB elimination.

## 1 Introduction

Tuberculosis (TB) remains one of the leading infectious causes of global morbidity and mortality (1). To mitigate this disease burden, the World Health Organization (WHO) established the End TB Strategy, aimed for a 20% reduction in incidence and a 35% cut in death numbers by 2020, relative to 2015. However, data from the Global Burden of Disease (GBD) study revealed a mere 6.26% decline in incidence and an 11.9% reduction in death numbers by that deadline-falling dramatically short of the established goals (2, 3). This significant shortfall highlights the urgent need to address a key driver of the epidemic: the vast reservoir of latent tuberculosis infection (LTBI).

Latent tuberculosis infection (LTBI) is defined as the presence of the pathogen within the host without manifestation of clinical tuberculosis, and in the absence of bacteriological or radiological evidence of active disease (4, 5). As reported in PLOS medicine, the burden is highly concentrated, with China and India alone accounting for approximately 350 million cases in 2014 (6). Though asymptomatic and non-infectious, it is identified by a positive tuberculin skin test (TST) or interferon-gamma release assay (IGRA), which indicates a host immune response (7). The clinical paradigm is shifting from a binary view of TB to a spectrum of infection, in which “early” LTBI represents a critical intervention window (8). The imperative for action is clear: without preventive therapy, 5–10% of individuals with LTBI will progress to active disease during their lifetime. Modeling studies confirm that even ceasing all new transmissions after 2015 would still result in a 2035 incidence of 16.5 per 100,000—far exceeding the End TB target of 10 per 100,000 (6), due entirely to reactivation of existing LTBI (7). This risk is profoundly inequitable, disproportionately affecting young children in households with active TB cases (with a two-year progression risk of up to 19%) and people living with HIV (with an annual progression risk of 5–10%) (9). Comorbidities such as diabetes further compound this risk (10). Therefore, LTBI is not a peripheral issue but the central frontier in the battle to eliminate TB.

China’s situation epitomizes this global challenge. While robust national systems like the Infectious Disease Reporting System (IDRS) and Tuberculosis Information Management System (TBIMS) have helped reduce incidence—by 20 per 100,000 from 2014 to 2021 (11)—the country continues to grapple with a massive latent pool, with prevalence estimated at 13–18% (12, 13). Progress is increasingly challenged by demographic and health transitions, including population aging, rising diabetes prevalence, and large-scale internal migration (14). These factors create a dynamic and complex epidemiological landscape, making the efficient targeting of LTBI interventions not just beneficial but essential for sustaining progress.

However, effective policy is hampered by a critical evidence gap. Research remains disproportionately focused on active TB, lacking comprehensive analyses of LTBI’s longitudinal trends, age-sex stratification, and future trajectories. This evidence gap is particularly acute in China, where fragmented data limit precision public health efforts. To bridge this gap, our study utilizes the GBD 2021 dataset to provide a 32-year (1990–2021), population-level characterization of LTBI across mainland China. We quantify age- and sex-specific prevalence and case numbers, benchmark China’s profile against global patterns, project future burden to 2050 using Bayesian age-period-cohort modeling, and identify optimal age windows for precision screening. This study aims to provide policymakers with actionable evidence to strategically target LTBI and accelerate China’s TB elimination agenda.

## 2 Methods

### 2.1 Data source and extraction

Data for this study were obtained from the Global Burden of Disease (GBD) 2021 study, which provides comprehensive, annually updated estimates of epidemiological measures for 371 diseases and injuries across 204 countries and territories. The GBD methodology integrates data from a wide range of diverse sources, including published literature, survey data, and administrative records, and employs rigorous modeling techniques to generate robust estimates. A detailed description of the GBD methodology has been published elsewhere (15). The geographical scope included global estimates and data for mainland China (which, in the GBD dataset, incorporates Hong Kong and Macao but excludes Taiwan). To align with TB epidemiology and policy objectives, the study population was stratified into five age groups: under-5, 5–14, 15–49, 50–69, and ≥70 years, adapting the standard GBD classification (16) to better reflect life-course stages relevant to TB risk. For age-period-cohort modeling, more granular 5-year age intervals (from 0–4 to 95 + years) were applied. Data were extracted and analyzed by sex and for both sexes combined.

### 2.2 Case definition and outcome metrics

The disease of interest was latent tuberculosis infection (LTBI). Because LTBI is an asymptomatic state, the GBD study does not estimate metrics such as incidence, mortality, years of life lost (YLLs), years lived with disability (YLDs), or disability-adjusted life years (DALYs) for this condition. Therefore, this study focused on prevalence as the primary burden metric. The specific metrics extracted for this analysis included age- and sex-stratified prevalence rates, age-standardized prevalence rates (ASPR) per 100,000 population, absolute case counts stratified by age, sex, year, and location, as well as the corresponding 95% confidence intervals (CIs) for all estimated metrics. Data on race and ethnicity were not available in the GBD database and were therefore excluded from our analysis. All diseases and injuries classifications in the GBD study adhere to the International Classification of Diseases, Tenth Revision (ICD-10).

### 2.3 Statistical analysis

To comprehensively assess the epidemiological trajectory, we employed three complementary analytical approaches. First, we calculated the Estimated Annual Percentage Change (EAPC) to quantify the average annual rate of change in the ASPR. Second, we applied joinpoint regression analysis to identify potential inflection points and segment-specific trends over the study period. Finally, to disentangle the independent effects of age, period, and birth cohort and project future disease burden, we implemented a Bayesian Age-Period-Cohort (BAPC) model (17).

#### 2.3.1 Estimated Annual Percentage Change (EAPC)

The EAPC provides a summary of the average annual rate of change over a continuous period (18). The EAPC was calculated by fitting a generalized linear model with a Gaussian distribution for the natural logarithm of the ASPR, using the calendar year as the predictor variable. The model is specified as ln (ASPR) = *α* + *β* (year) + *ε*, where *β* is the regression coefficient. The EAPC was then derived from the model’s regression coefficient (*β*) using the formula EAPC = 100 × [exp(β) − 1]. The 95% CI was also calculated from the model. A trend was considered statistically significant if its 95% CI did not include zero.

#### 2.3.2 Joinpoint regression analysis

For a more nuanced examination of temporal patterns, we conducted joinpoint regression analysis using the Joinpoint Regression Program (Version 5.3.0; National Cancer Institute, USA). This method identifies significant inflection points(joinpoints) in time-series data, which allows the study period to be segmented into distinct trend phases. The model was fitted to the natural logarithm of prevalence against calendar year, allowing a maximum of six joinpoints over the 32-year period and requiring at least three observations between consecutive points. Model selection utilized a permutation test (*α* = 0.05; 4,499 Monte Carlo simulations). For each identified segment, we calculated the Annual Percent Change (APC) and its 95% confidence interval. The Average Annual Percent Change (AAPC) with its 95% confidence interval was computed to summarize the overall trend across the entire study period. When no joinpoints are identified, the model’s output is equivalent to the EAPC estimate.

#### 2.3.3 Bayesian Age–Period–Cohort (BAPC) modeling and forecasting

##### 2.3.3.1 Statistical analysis: Bayesian age-period-cohort modeling

The number of observed LTBI cases within each age-period cell, denoted as $Y_{\mathrm{ij}}$ , was modeled using a Poisson distribution:


$$Y_{\mathrm{ij}}∼\text{Poisson}(\lambda_{\mathrm{ij}}\cdot {\mathrm{Pop}}_{\mathrm{ij}})$$

where $\lambda_{\mathrm{ij}}$ represents the underlying prevalence rate for the i -th age group and j -th period, and $\mathrm{Po}p_{\mathrm{ij}}$ is the corresponding person-years at risk, which was included in the model as an offset term to account for differences in population size.

The linear predictor was specified on the logarithmic scale as:


$$\log(\lambda_{\mathrm{ij}})=\mu +\alpha_{i}+\beta_{j}+\gamma_{k}$$

Here, μ is the model intercept, representing the overall baseline log-rate. The term $\alpha_{i}$ denotes the age effect for the i -th age group and captures the typical life-course pattern of LTBI risk. The term $\beta_{j}$ denotes the period effect for the j -th period, reflecting population-wide temporal changes driven by factors such as public health interventions or environmental shifts. The term $\gamma_{k}$ denotes the cohort effect for the k -th birth cohort (corresponding to individuals born in period *j* minus age *i*), indicating long-term risk variations across generations.

##### 2.3.3.2 Prior specifications and identifiability constraints

The intrinsic identifiability issue in APC models, which arises from the perfect linear dependency between age, period, and cohort (Age + Cohort ≈ Period), was addressed by imposing biologically plausible smoothing constraints via the choice of prior distributions.

Second-order random walk (RW2) priors were assigned to the age ( $\alpha_{i}$ ), period ( $\beta_{j}$ ), and cohort ( $\gamma_{k}$ ) effects. This prior assumes that the second-order differences of the effect parameters follow a normal distribution with a mean of zero, which penalizes abrupt changes between adjacent groups and yields smoother trend estimates. This flexibility allows the model to capture gradual temporal shifts and generational changes effectively.

A diffuse normal prior, $\mu ∼\text{Normal}(0,10^{6})$ , was placed on the intercept, indicating minimal prior information regarding the overall baseline rate. The precision parameters ( $\tau_{\alpha },\tau_{\beta },\tau_{\gamma }$ ) governing the smoothness of the RW2 priors were assigned weakly informative Gamma priors, Gamma(0.5,0.0005) , which allows the data to primarily drive the degree of smoothing.

##### 2.3.3.3 Model estimation and inference

The posterior distributions of the model parameters were estimated using the Integrated Nested Laplace Approximation (INLA) (19). INLA offers a computationally efficient and accurate alternative to traditional Markov Chain Monte Carlo (MCMC) methods by directly approximating the marginal posterior distributions, thereby circumventing the need for convergence diagnostics and lengthy simulation times.

Parameter estimates for age, period, and cohort effects are reported as the median of their marginal posterior distributions. The associated uncertainty for each estimate is quantified using the 95% Highest Posterior Density (HPD) interval, which represents the most credible interval containing the true parameter value with a probability of 0.95.

##### 2.3.3.4 Sensitivity analysis

To assess the robustness of our findings to the choice of prior distributions for the precision parameters, we conducted a sensitivity analysis. An alternative model was specified, replacing the Gamma(0.5,0.0005) prior with a weakly informative Half−Normal(0,1) prior. A comparative analysis of the estimated age, period, and cohort effect curves—along with their 95% HPD intervals—from the primary and alternative models revealed no substantive differences in key conclusions, such as the location of trend inflection points or effect peaks. This consistency confirms that our primary results are robust to the specific choice of weakly informative prior.

BAPC analyses were performed using the BAPC package in R (version 4.2.3) and JD_GBDR (V2.24) (18).

#### 2.3.4 Projections of LTBI prevalence trends, 2022–2050

Using the fitted BAPC model, we projected the age-standardized prevalence rates of LTBI in China and Globally through 2050. Projections were based on the extrapolation of the estimated period and cohort effects, combined with country-specific population forecasts (by age and sex) provided by the Institute for Health Metrics and Evaluation (IHME).

## 3 Results

### 3.1 Global and mainland China LTBI prevalence trends, 1990–2021

Globally, LTBI prevalence declined from 1990 to 2021 (EAPC = −0.97, 95%CI -1.02 to −0.92), yet the total number of cases rose by 19.34%. Mainland China maintained a high burden and experienced a slower rate of decline (EAPC = −0.39, 95%CI -0.52 to −0.26). Conversely, the estimated number of cases in China increased substantially. In 2021, China ranked 59th globally in LTBI prevalence yet had the highest absolute number of cases.

**Figure 1:**
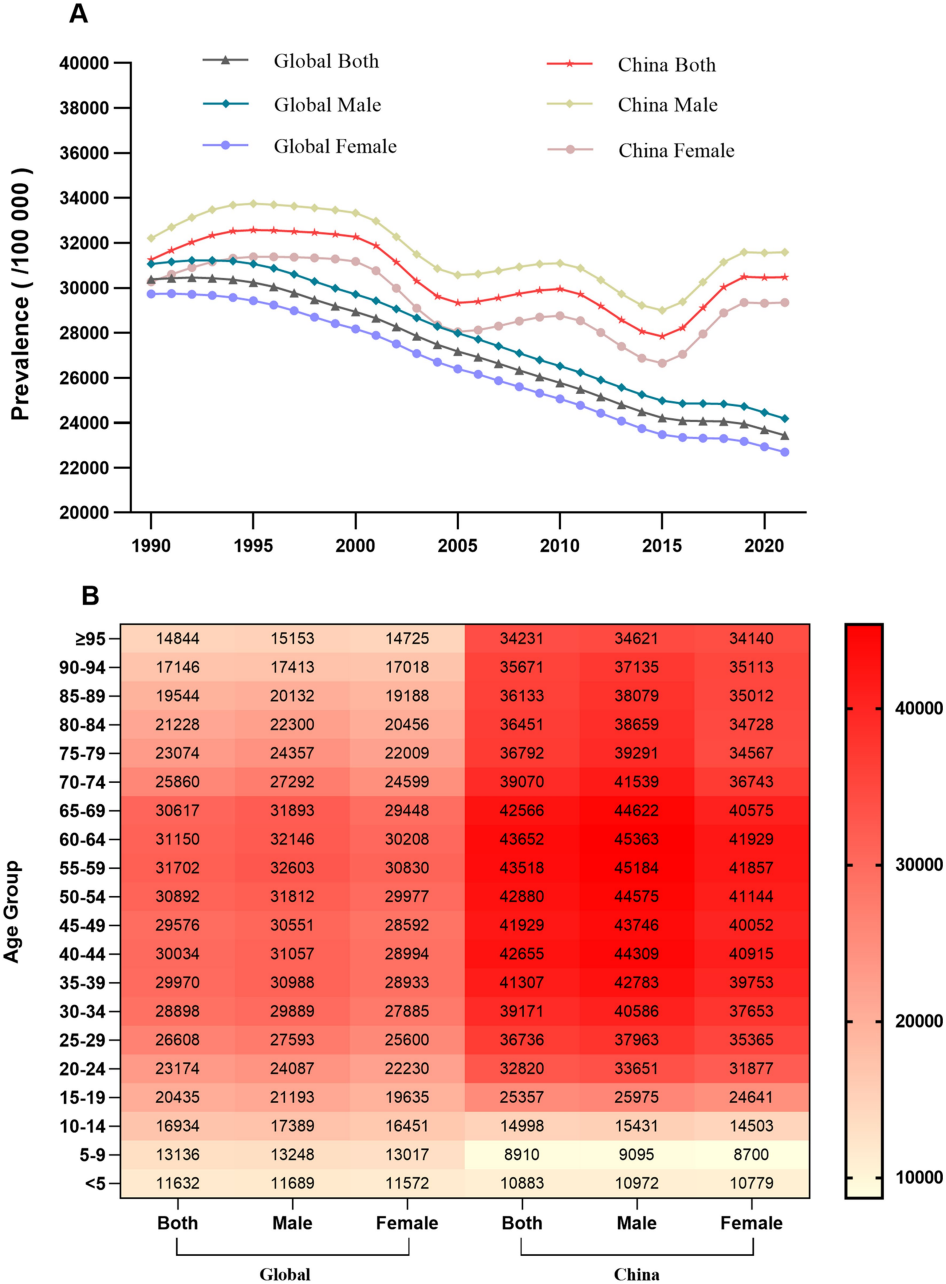
Comprehensive analysis of the prevalence of latent tuberculosis infection (LTBI), 1990–2021. **(A)** Time trends of the age-standardized prevalence of LTBI for males and females in China and the global average. **(B)** Heatmap of LTBI prevalence rates (per 100,000 population) in 2021, stratified by sex and 5-year age groups, for China and the global average. Colour intensity indicates the prevalence rate, from low (light) to high (dark).

Regarding gender disparities, prevalence was consistently higher among males, both globally and in China. Globally, age-specific prevalence peaked in the 50–59 years age group, whereas in China the peak occurred later, in the 60–64 years age group. While the prevalence in China was lower than the global average for age groups under 14 years, it surpassed the global average in all older age groups (Table 1; Figure 1).

**Table 1:** Epidemiological characteristics of latent tuberculosis infection in global and mainland China, 1990–2021.

Characteristics	1990		2021		1990–2021	
	Prevalence cases (*10^9^)	Prevalence/100,000	Prevalence cases (*10^9^)	Prevalence/100,000	Case change (%)	EAPC
Global	1.59	30384.69	1.90	23435.37	19.34	−0.97(−1.02, −0.92)
China	0.37	31257.74	0.49	30484.35	33.28	−0.39(−0.52, −0.26)
Global sex						
Male	0.82	31057.82	0.98	24188.67	20.06	−0.94(−0.99, −0.89)
Female	0.77	29733.78	0.92	22685.63	18.58	−0.99(−1.04, −0.95)
China sex						
Male	0.20	32214.55	0.26	31580.89	32.57	−0.37(−0.49, −0.25)
Female	0.17	30268.04	0.23	29349.50	34.08	−0.41(−0.55, −0.27)
Global age						
≤5	0.11	18023.81	0.08	11632.41	−31.48	−1.51(−1.58, −1.43)
5–14	0.25	22113.52	0.20	15006.68	−17.92	−1.32(−1.39, 1.25)
15–49	0.93	34137.72	1.06	26731.20	14.07	−0.92(−0.97, 0.86)
50–69	0.24	35887.41	0.45	31119.93	82.66	−0.65(−0.71, −0.58)
>70	0.06	29992.40	0.12	23275.39	89.92	−0.97(−1.03, −0.90)
China age						
<5	0.02	15347.90	0.01	10882.56	−50.74	−1.61(−2.28, −0.93)
5–14	0.03	15243.01	0.02	11794.00	−31.84	−0.81(−1.28, −0.34)
15–49	0.24	36451.64	0.25	37878.41	3.36	−0.17(−0.30, −0.04)
50–69	0.06	39623.27	0.16	43148.93	169.60	−0.17(−0.31, −0.01)
>70	0.02	40548.51	0.04	37659.26	195.24	−0.45(−0.58, −0.32)

### 3.2 Temporal trends of AAPC in LTBI prevalence

Joinpoint regression provided a more nuanced and detailed analysis of temporal patterns than the EAPC, revealing several distinct trend periods.

From 1990 to 2021, while global AAPC showed a consistent decline (−0.84, 95% CI -0.88 to −0.81), mainland China’s prevalence exhibited pronounced fluctuations, although the overall trend showed a slight decline (−0.09, 95% CI -0.13 to −0.05). An increasing trend was observed during 1990–1994, 2005–2010, and 2015–2019, while a decreasing trend was observed during 2000–2005 and 2010–2015.

**Figure 2:**
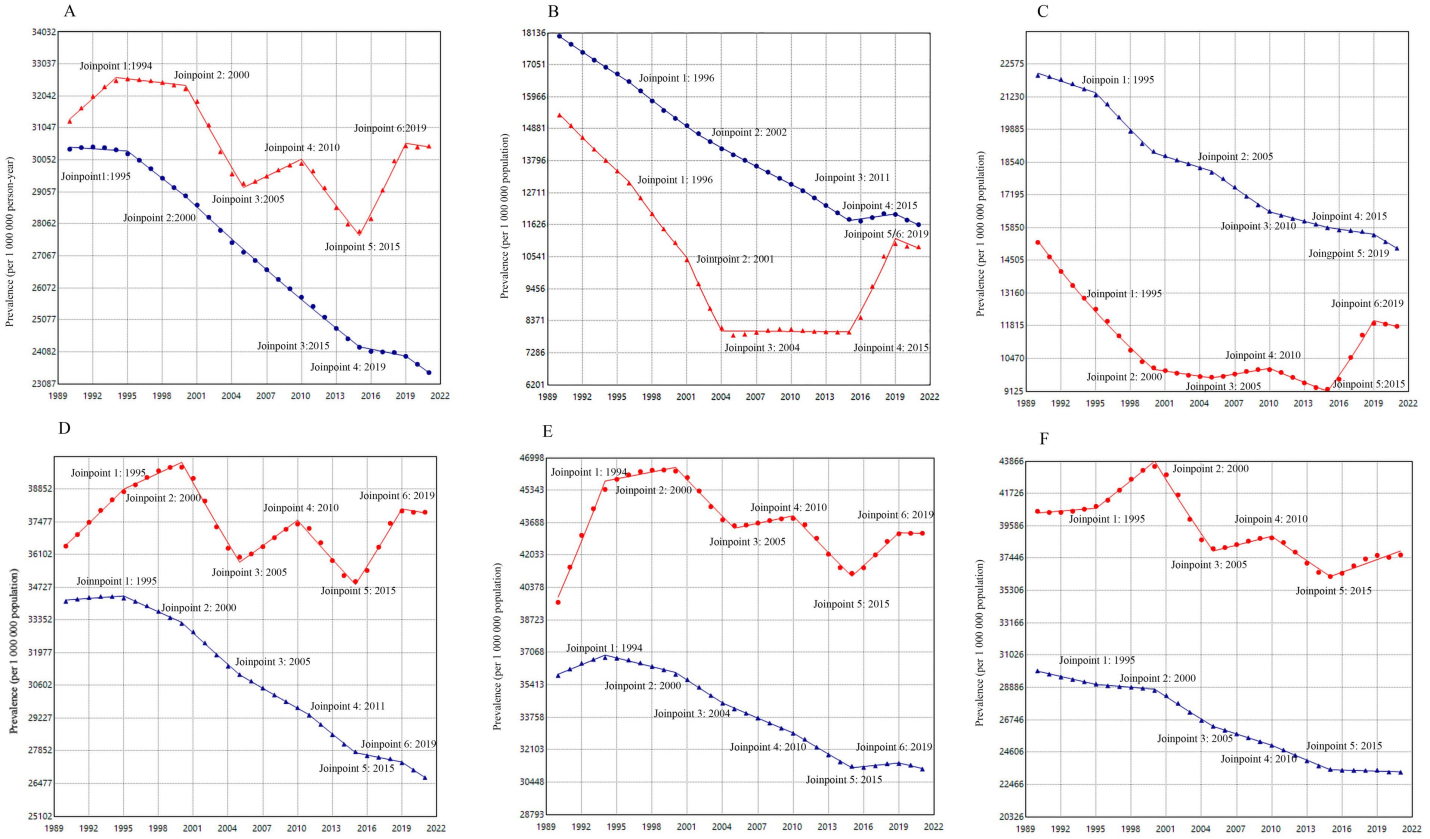
Joinpoint regression comparison of prevalence rates between mainland China and the global, 1990–2021. **(A)** Age-standardized prevalence **(B)** ≤ 5 years **(C)** 5 ~ 14 years **(D)** 15 ~ 49 years **(E)** 50 ~ 69 years **(F)** ≥ 70 years. In all panels, the blue lines represent the global average and the red lines represent mainland China. The trends are characterized by segmented linear regressions, with each joinpoint indicating a significant change in the trend.

According to the best-fit solution, age-stratified analyses revealed a downward trend across all age groups globally over the past three decades. The magnitude of the AAPC reduction first decreased with age, and then increased, reaching its lowest point in the 50–69 age group (−0.46, 95% CI -0.49 to −0.43). In mainland China, the magnitude of AAPC changes was consistently smaller than the global level across all age groups, with substantial variation between groups. The 15–49 and 50–69 age groups displayed an overall upward trend, while other age groups exhibited downward trends (Figure 2; Table 2).

**Table 2:** Average Annual Percentage Change (AAPC) in latent tuberculosis infection prevalence globally and in mainland China, 1990–2021.

Age group	Cohort	AAPC	Lower CI	Upper CI	*P*
Age-standardized	Global	−0.84*	−0.88	−0.81	< 0.01
Age-standardized	China	−0.09*	−0.13	−0.05	< 0.01
<5	Global	−1.40*	−1.45	−1.36	< 0.01
<5	China	−1.13*	−1.37	−0.87	< 0.01
5 ~ 14	Global	−1.25*	−1.33	−1.17	< 0.01
5 ~ 14	China	−0.82*	−0.91	−0.74	< 0.01
15 ~ 49	Global	−0.79*	−0.82	−0.77	< 0.01
15 ~ 49	China	0.12*	0.07	0.16	< 0.01
50 ~ 69	Global	−0.46*	−0.49	−0.43	< 0.01
50 ~ 69	China	0.25*	0.17	0.34	< 0.01
≥70	Global	−0.81*	−0.84	−0.78	< 0.01
≥70	China	−0.21*	−0.31	−0.09	< 0.01

* *p* < 0.05.

### 3.3 Age-period-cohort analysis of LTBI prevalence

**Figure 3:**
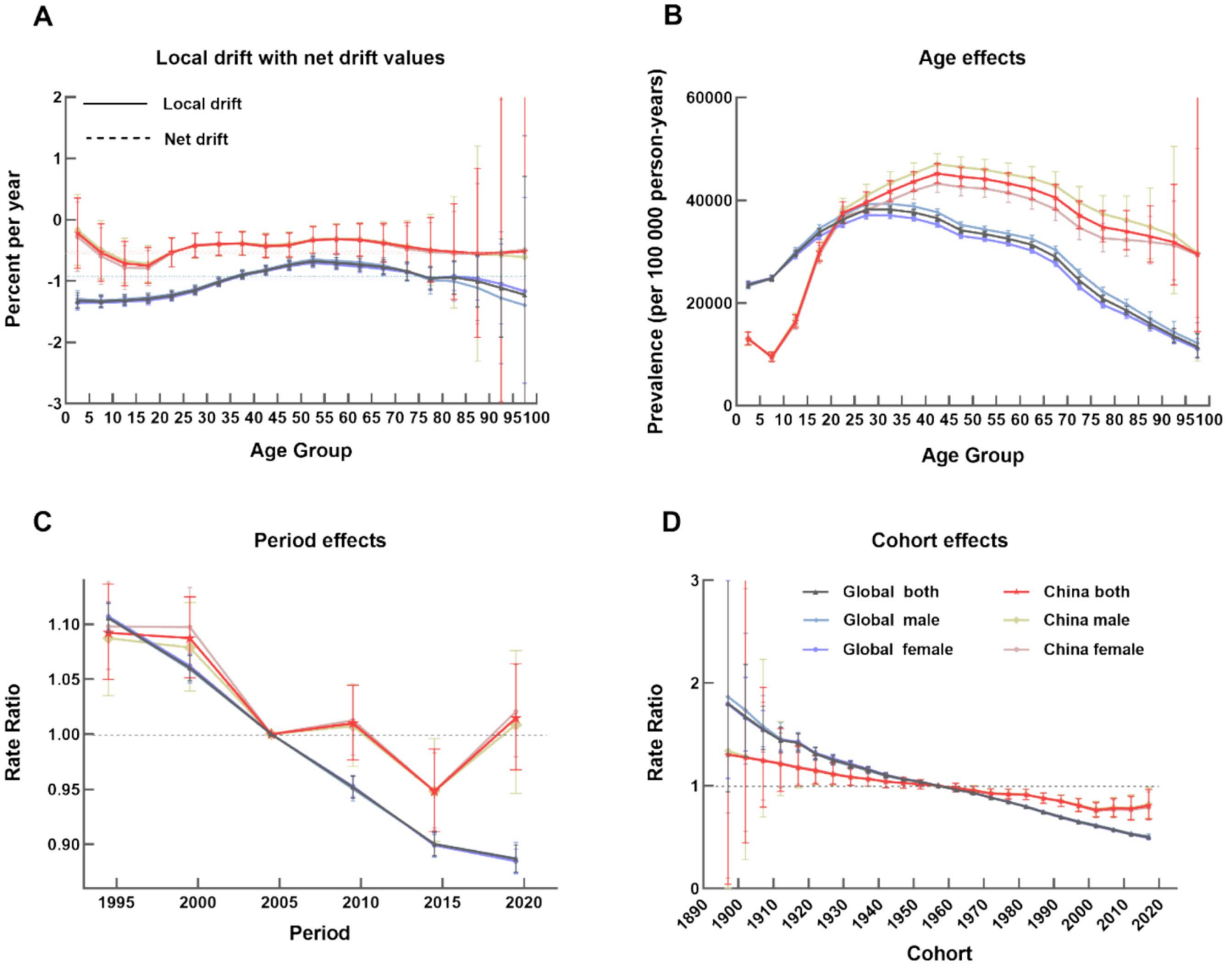
Age-period cohort distribution of average age-standardized prevalence in global and mainland China, 1990–2021. **(A)** Local drifts, showing Estimated Annual Percentage Change (EAPC) by age group. Net drift, representing overall annual percentage change in prevalence, after removing the influence of age structure. **(B)** Age effects, representing the inherent risk across the lifespan, independent of period and cohort influences. **(C)** Period effects, reflecting the risk changes attributable to specific time points that affect all age groups simultaneously. **(D)** Cohort effects, indicating the lifetime risk associated with different birth cohorts. In all panels, the blue lines represent the global average and the red lines represent mainland China. Shaded areas denote 95% confidence intervals.

The APC analysis revealed a downward trend both globally and in mainland China (Figure 3). The global net drift was −0.94% (95% CI:-1.00% to −0.87%), compared with a slower decline in China (−0.44, 95% CI:-0.70% to −0.18%). Globally, females showed a faster decline than males globally (−0.95% vs. -0.93%); this pattern was also observed in China (−0.45% vs. -0.43%). Contrary to the consistent downward trend seen worldwide, the epidemiological profile of LTBI in mainland China was distinct and more complex, marked by a persistently high burden with signs of stalled progress.

#### 3.3.1 Age effects

In China, the risk of LTBI increased with age, peaking between 25–60 years - a much longer duration than the global peak (20–29 years) - and this effect was more pronounced in males. The risk curves for China and the globe intersected at approximately 22.5 years of age, suggesting a lower risk for children in China compared to the global average. This pronounced plateau of elevated risk among the middle-aged population represents a substantial pool of latent infection, which serves as a considerable reservoir for potential future tuberculosis reactivation.

#### 3.3.2 Period effects

While period-related risks declined globally, China experienced considerable fluctuations. The risk was higher than the reference period (2000–2004) before 2004, then generally declined but saw increases during 2005–2010 and 2015–2019, ultimately exceeding the reference level again.

#### 3.3.3 Cohort effects

Compared with the 1957–1962 reference cohort, the risk of LTBI declined in subsequent birth cohorts both globally and in China, though the decline was less pronounced in China. A slight rebound in risk was observed in the 2017–2022 birth cohort in China.

### 3.4 Future projections for LTBI (2022–2050)

**Figure 4:**
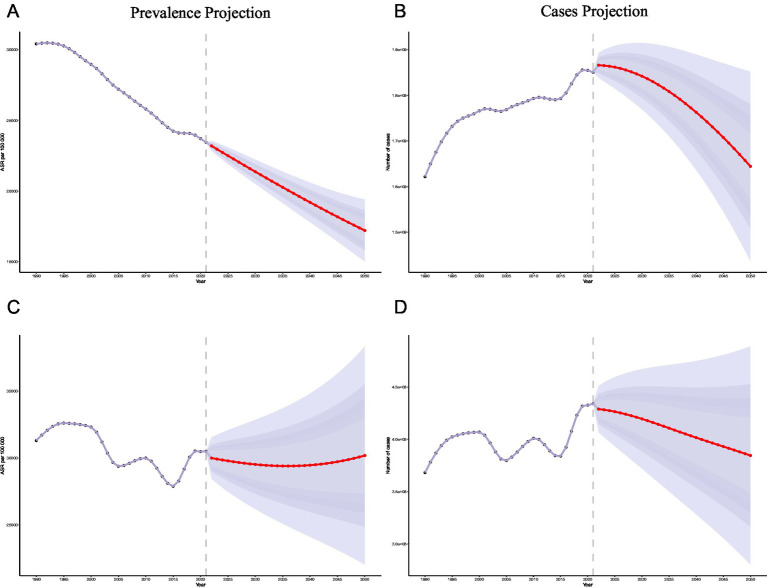
Projection of LTBI prevalence in 2050 in global and mainland China **(A)** Projections of global LTBI prevalence **(B)** Projections of global LTBI infection cases **(C)** Projections of China LTBI prevalence **(D)** Projections of China LTBI infection cases. All projections are based on the Bayesian Age-Period-Cohort (BAPC) model and historical data from 1990 to 2021. Shaded areas represent the 95% confidence intervals or prediction intervals.

The BAPC model projects a continued steady decline in global LTBI prevalence over the next three decades, expected to reach a minimum by 2050 (17,217.35 ± 1,112.60). In mainland China, however, the prevalence is projected to fluctuate. While the model anticipates an initial decline from 2022 to 2036 (29,396.35 ± 2,309.42), it predicts a subsequent rebound, with the prevalence reaching 30,184.31 by 2050. Globally, the estimated number of LTBI cases is projected to peak in 2036 (1.80 × 10^9^ ± 0.53 × 10^9^) before declining to 1.64 × 10^9^ ± 1.06 × 10^9^ by 2050. In mainland China, this peak is projected to occur earlier, in 2029 (0.42 × 10^9^ ± 0.02 × 10^9^), followed by a decline to 0.38 × 10^9^ ± 0.05 × 10^9^ by 2050. These projections underscore the need for continuous monitoring and adaptive control measures to prevent a future resurgence of LTBI in China (Figure 4).

## 4 Discussion

Tuberculosis (TB) remains a substantial contributor to the global burden of infectious diseases (20). Against this backdrop, our study delineates the evolving epidemiology of latent tuberculosis infection (LTBI) in mainland China from 1990 to 2021, situating it within broader global patterns. We observe that while global LTBI prevalence has followed a steady downward trajectory, mainland China has experienced considerable fluctuation over the same period. Given that TB risk and appropriate interventions vary considerably across the life course (21, 22), we further present a detailed, age- and sex-stratified analysis of these temporal shifts in LTBI burden.

The incidence of childhood TB is a critical indicator of recent community transmission and reflects the overall effectiveness of TB control programs (23). Consistent with WHO estimates that children under 15 account for approximately 12% of all TB cases (24, 25), our findings indicate that although a slight rebound in risk was observed for the 2017–2022 birth cohort, the overall burden of LTBI among children in mainland China remains below the global average. This declining trend aligns with national surveillance data, which show a continuous decrease in reported pulmonary TB incidence among children under 15 since 1997 conducted by the Tuberculosis Prevention and Control Project of the Chinese Center for Disease Control and Prevention (26). Despite this progress, childhood TB control continues to face substantial challenges. Due to their immature immune systems, infants and young children are more susceptible to developing severe forms of TB, such as disseminated disease and tuberculosis meningitis (27). Diagnosis is particularly challenging in this population, as pediatric TB is often paucibacillary, presents with nonspecific or subtle radiological findings, and molecular assays have limited sensitivity, making clinical assessment essential (28). Furthermore, widespread BCG vaccination in China (29) reduces the predictive utility of both tuberculin skin tests (TST). Epidemiological reporting on childhood TB remains incomplete in mainland China (30), and operational gaps persist. For instance, a study from Shanghai (31) revealed that only 33.3% of child contacts of TB patients underwent screening, and knowledge among primary care providers regarding LTBI progression risk, preventive treatment options, and treatment eligibility criteria was generally low (7). Moving forward, a comprehensive risk–benefit framework is needed to strengthen pediatric TB control. Priorities should include improving risk stratification to identify high-risk child contacts for LTBI screening, developing of child-friendly drug formulations, implementation of directly observed therapy, and the establishment of family-centered support systems to enhance both screening uptake and treatment completion among children (6).

Our analysis reveals a distinct and protracted age-specific pattern of LTBI in mainland China compared to the global profile. While prevalence among those under 14 years old was lower than the global average, it consistently surpassed global levels in all older age groups. Notably, the peak risk period in China was substantially prolonged, spanning the 25–60 age range, in contrast to the more concentrated global peak observed at 20–29 years. This prolonged high-risk window, encompassing a vast segment of the working-age and older adult population, demands a recalibration of targeted intervention strategies in the country. Despite significant national investments in TB control—evidenced by expanded diagnostic (32, 33) and treatment coverage (34)—ensuring equitable and effective implementation across all demographic groups and regions remains a critical challenge. The LTBI burden was consistently higher and more sustained among males, a disparity likely driven by a combination of sociobehavioral factors. These factors include higher rates of smoking rates, greater social mobility, and overrepresentation in high-exposure occupations such as mining and construction (35). Furthermore, internal migrant populations, moving from rural to urban areas, represent a vulnerable group due to crowded living conditions and fragmented health coverage, creating potential hotspots for TB transmission and challenges for LTBI management (36). Compounding these challenges is China’s rapidly aging population. The proportion of the population aged ≥65 years rose from 6.5% in 1997 to 14.9% in 2022 (37), and is projected to exceed 20% by 2035 (38). Immunosenescence, combined with a high prevalence of comorbidities such as diabetes and malnutrition significantly increases the vulnerability of older adults to TB reactivation and severe disease outcomes (39). This demographic shift poses a formidable challenge to national TB control, necessitating the urgent scale-up of active case-finding and optimized management strategies tailored for the elderly to mitigate the growing burden of TB in this population.

The COVID-19 pandemic triggered a massive reallocation of global public health resources, diverting critical attention and funding away from longstanding priorities like tuberculosis (TB). In 2020 alone, research funding for COVID-19 surged to over US$100 billion, starkly overshadowing the meager US$0.9 billion allocated to TB research (38, 40). This dramatic shift in funding priorities has likely compromised the continuity and effectiveness of TB control programs worldwide. In China, the pandemic response led to a substantial redeployment of healthcare personnel and resources from the TB control system to COVID-19 efforts. Concurrently, concerns about virus transmission reduced population mobility. As Fei et al. (41) reported, these factors contributed to delays in TB case reporting and interruptions in patient follow-up within China’s TB control system. Our study indicates a decline in LTBI prevalence after 2019, a trend observed both globally and in China. This decrease could be superficially attributed to reduced transmission resulting from infection control measures and lowered mobility. However, it is crucial to recognize that the large-scale diversion of diagnostic and human resources to COVID-19 screening almost certainly weakened the capacity for active LTBI detection. This likely artificial depression in detected prevalence highlights a critical vulnerability: emergency pandemic responses can undermine essential disease surveillance and control programs, creating a false sense of security while potentially allowing a hidden burden of infection to accumulate.

Notably, subclinical TB plays a more significant role in transmission than previously acknowledged. Aerosol studies indicate that even individuals with paucibacillary or undiagnosed TB can be highly contagious, highlighting the limitations of a rigid active-versus-latent framework (42). Treating LTBI reduces the risk of progression to active disease by 60–90% (43), underscoring the importance of screening and tuberculosis preventive treatment (TPT) for close contacts, as endorsed by both the WHO and Chinese health guidelines.

However, universal LTBI screening and TPT are impeded by high costs, operational challenges, and potential adverse drug reactions. Cost-effectiveness analysis is therefore essential for guiding targeted strategies. A study from South Korea demonstrated that targeting adults aged 35–64 yielded the greatest reduction in TB incidence (44). Shorter regimens, such as the 3HP regimen (3 months of weekly rifapentine and isoniazid), have shown superior adherence compared to longer courses in a multicenter cluster-randomized trial in China (45). Nonetheless, adverse events remain a leading cause of treatment discontinuation. New vaccine strategies tailored to uninfected individuals or those with LTBI represent a potentially cost-effective approach and are a major focus of current research (44). Additionally, TPT has proven highly effective among high-risk groups - such as people living with HIV and household contacts of DR-TB patients - though resource constraints complicate implementation (46). The WHO provides differentiated TPT recommendations based on a country’s TB incidence and income level. In China, LTBI management is integrated into the national TB control strategy, which prioritizes high-risk groups such as HIV-positive individuals, children under 5, and students, thereby maximizing resource efficiency and mitigating transmission (47).

### 4.1 Implications for policy and practice

Our findings underscore the urgent need to reinvigorate tuberculosis (TB) control efforts in China. While the Bayesian age-period-cohort model projects fluctuations in latent tuberculosis infection (LTBI) prevalence over the coming decades, recent advances in diagnostics and therapeutics offer a tangible opportunity to alter this trajectory. Achieving the WHO’s End TB Strategy targets will require a dual approach: ensuring effective management of active TB cases while systematically preventing progression from LTBI to active disease (16, 48). Key strategic priorities should include:

#### 4.1.1 Strengthening LTBI screening

Expanding access to accurate and cost-effective screening tools for high-risk groups—such as close contacts of TB patients (43), people living with HIV, and individuals with comorbidities—is essential. Following the example of Mexico’s successful integration of TB screening into diabetes clinics (49), China could similarly leverage chronic disease management platforms for targeted case-finding. Domestically, the newly released National Tuberculosis Prevention and Control Plan (2024–2030) (50) designates rapid molecular diagnostics as the primary method for TB diagnosis in designated hospitals, a policy expected to significantly improve case detection efficiency.

#### 4.1.2 Optimizing treatment and prophylaxis

The pipeline for new TB vaccines currently includes 16 candidates in clinical trials. Although progress has been incremental, modeling studies show that a vaccine with 60% efficacy in adolescents and adults that provides 10 years of protection could avert 17 million TB cases between 2024 and 2050 (51). Concurrently, traditional Chinese medicine has explored syndrome pattern-based treatment approaches—categorized as “staged differentiation” and “syndrome differentiation”—aimed at early LTBI intervention, immune homeostasis regulation, and risk reduction (52). These approaches, combined with ongoing global efforts to develop shorter, better-tolerated LTBI regimens and promote patient-centered care (53), could provide a multifaceted strategy for ending the TB epidemic.

#### 4.1.3 Enhancing surveillance systems

Implementing robust, real-time surveillance, as demonstrated by South Korea’s integrated national program, is critical for monitoring epidemiological trends, identifying high-risk populations, and evaluating intervention impact, as demonstrated by South Korea’s integrated national program (54). For instance, given the persistently high burden projected for the 50–69 age group, integrating LTBI screening into this demographic’s annual health check-ups could be a targeted, feasible strategy.

#### 4.1.4 Addressing social determinants

Sustained investment in socioeconomic interventions—such as poverty reduction, improved housing conditions, and mitigation of other structural risk factors—is fundamental to long-term TB control. Reducing these inequities will directly decrease transmission risk and lessen the disease’s disproportionate impact on vulnerable communities.

### 4.2 Limitations

This study has several limitations that must be considered when interpreting the results. First, the LTBI estimates are derived from modeled GBD data rather than direct surveillance data, which introduces inherent uncertainty. In China, the national TB surveillance system focuses primarily on active pulmonary tuberculosis, while systematic reporting and management of LTBI remain underdeveloped. These gaps in underlying data may affect the accuracy of the GBD burden estimates. Second, our analysis did not account for several important individual-level risk factors—including occupational exposure, smoking, HIV coinfection, and BCG vaccination history—which could influence LTBI progression and thus limit the precision of our estimates. Third, while this study describes epidemiological trends in LTBI, it does not formally examine the specific drivers behind these patterns, such as changes in TB control policies or healthcare-seeking behaviors. Further research is needed to explore these underlying factors and help tailor more effective interventions.

## 5 Conclusion

Mainland China faces a substantial LTBI burden that poses a significant challenge to achieving its TB elimination targets. Our findings highlight the need for strengthened and sustained efforts to prevent, diagnose, and treat LTBI, with a particular focus on high-risk populations and high-burden regions. By addressing the social determinants of health and implementing comprehensive control strategies, China can make significant strides toward ending the TB epidemic within its borders. Future research should incorporate large-scale, prospective cohort studies that combine data on individual-level risk behaviors with biological sampling to more accurately delineate the epidemiological landscape of LTBI and generate robust evidence for developing highly targeted interventions.

## Data Availability

Publicly available datasets were analyzed in this study. This data can be found at: the datasets generated during and/or analyses during the current study are available in the Global Health Data Exchange (GHDx) platform (http://ghdx.healthdata.org/gbd-results-tool).
